# HPV-18 E6 Oncoprotein and Its Spliced Isoform E6*I Regulate the Wnt/β-Catenin Cell Signaling Pathway through the TCF-4 Transcriptional Factor

**DOI:** 10.3390/ijms19103153

**Published:** 2018-10-13

**Authors:** J. Omar Muñoz-Bello, Leslie Olmedo-Nieva, Leonardo Josué Castro-Muñoz, Joaquín Manzo-Merino, Adriana Contreras-Paredes, Claudia González-Espinosa, Alejandro López-Saavedra, Marcela Lizano

**Affiliations:** 1 Unidad de Investigación Biomédica en Cáncer, Instituto Nacional de Cancerología-Instituto de Investigaciones Biomédicas, Universidad Nacional Autónoma de México, Mexico City 14080, Mexico; omarmube@gmail.com (J.O.M.-B.); leslie_azul25@hotmail.com (L.O.-N.); joscasmunoz@gmail.com (L.J.C.-M.); adrycont@yahoo.com.mx (A.C.-P.); alexlosaav@gmail.com (A.L.-S.); 2 Programa de Doctorado en Ciencias Biomédicas, Universidad Nacional Autónoma de México, Mexico City 04510, Mexico; 3 CONACyT-Instituto Nacional de Cancerología, Mexico City 14080, Mexico; jmanzome@conacyt.mx; 4 Departamento de Farmacobiología, Centro de Investigación y Estudios Avanzados del Instituto Politécnico Nacional, Sede sur, Mexico City 14330, Mexico; cgonzal@cinvestav.mx; 5 Departamento de Medicina Genómica y Toxicología Ambiental, Instituto de Investigaciones Biomédicas, Universidad Nacional Autónoma de México (UNAM), Mexico City 04510, Mexico

**Keywords:** HPV-18 E6, HPV-18 E6*I, TCF-4 transcription factor, Wnt/β-catenin signaling

## Abstract

The Wnt/β-catenin signaling pathway regulates cell proliferation and differentiation and its aberrant activation in cervical cancer has been described. Persistent infection with high risk human papillomavirus (HR-HPV) is the most important factor for the development of this neoplasia, since E6 and E7 viral oncoproteins alter cellular processes, promoting cervical cancer development. A role of HPV-16 E6 in Wnt/β-catenin signaling has been proposed, although the participation of HPV-18 E6 has not been previously studied. The aim of this work was to investigate the participation of HPV-18 E6 and E6*I, in the regulation of the Wnt/β-catenin signaling pathway. Here, we show that E6 proteins up-regulate TCF-4 transcriptional activity and promote overexpression of Wnt target genes. In addition, it was demonstrated that E6 and E6*I bind to the TCF-4 (T cell factor 4) and β-catenin, impacting TCF-4 stabilization. We found that both E6 and E6*I proteins interact with the promoter of *Sp5*, in vitro and in vivo. Moreover, although differences in TCF-4 transcriptional activation were found among E6 intratype variants, no changes were observed in the levels of regulated genes. Furthermore, our data support that E6 proteins cooperate with β-catenin to promote cell proliferation.

## 1. Introduction

The Wnt signaling pathway regulates a variety of processes, including cell proliferation and differentiation [1]. Briefly, in the off-state of the canonical pathway, the effector protein, β-catenin, is associated to a multiprotein complex that promotes its phosphorylation in specific residues in a GSK3β (Glycogen synthase kinase 3β) and CK1 (Casein kinase 1) kinase-dependent fashion. Those residues are recognized by the ubiquitin-ligase β-TrCP, allowing β-catenin ubiquitylation and subsequent degradation via the proteasome, whilst in the nucleus, the co-repressor, Groucho/TLE (Transducin-like enhancer), suppresses transcriptional activation through the inhibition of TCF/LEF (Lymphoid enhancer binding factor) transcriptional factors. In the on-state, the Wnt ligands bind to the Frizzled receptor and the LRP 5/6 co-receptor, which dimerize, leading to disassembly of the destruction complex. Subsequently, β-catenin is released in the cytoplasm and translocated into the nucleus, where it binds to TCF/LEF and replaces the repressor protein, Groucho/TLE [2]. This event induces TCF/LEF transcriptional activation and expression of genes, such as *Axin2*, *Jun*, *Myc*, *Ccnd1*, and *Sp5* (Specificity protein transcription factor 5) [3]. It has been demonstrated that alterations in the Wnt cell signaling pathway contribute to the development of several types of cancer [4], including colorectal [5], hepatocarcinoma [6], breast [7], and HPV-related cancers [8,9,10].

The persistent infection with high risk human papillomavirus (HR-HPV) is the main risk factor associated to cervical cancer development [11]. HPV-16 and HPV-18 are the most prevalent types, found in almost 70% of cervical cancer cases worldwide [12]. HR-HPV transformation capacity is mainly due to the overexpression of the E6 and E7 viral oncoproteins, which interact with many cellular proteins, thus affecting their functions [13]. E6 is implicated in the modulation of several cell signaling pathways involved in cell adhesion, proliferation, and apoptosis, such as RAF (Rapidly accelerated fibrosarcoma)/MEK (MAPK/ERK kinase)/ERK (Extracellular signal-regulated kinase) and PI3K (Phosphoinositide 3 kinase), among others [14].

The E6–E7 open reading frames (ORFs) contain spliced donor and acceptor sites, highly conserved among the HR-HPV. Those sites are recognized by the spliceosome complex, promoting the removal of a small intron and the generation of a premature stop codon, giving place to short forms of E6, termed E6*. In HPV-16, at least four isoforms of E6* (I–IV) have been identified, whereas in HPV-18, only one has been reported hitherto, termed E6*I [15]. Although these E6 small isoforms are highly expressed in premalignant lesions and cervical cancer biopsies [16,17], their functions are poorly understood [18].

The abnormal activation of the Wnt cell signaling pathway has been reported in HPV-related tumors [8,19,20]. In cervical tumor biopsies and HPV positive cell lines, β-catenin is mainly located in the cytoplasm and nucleus, while in normal tissue, it is mainly distributed at the cell membrane [9,20]. In vitro assays have demonstrated that HPV-16 E6 induces TCF-4 transcriptional activation, whereas β-catenin is not stabilized. Moreover, E6AP ubiquitin ligase contributes to the increase in the TCF transcriptional activation mediated by E6, in a proteasome-dependent manner, without affecting β-catenin levels [21,22]. 

No interactions of HPV-18 E6 protein with members of the Wnt activation complex (TCF-4, β-catenin) have been identified so far, and the mechanisms by which E6 induces TCF transcriptional activation are poorly understood. Moreover, the effect of E6* proteins in this pathway remains unknown. Therefore, the aim of this study was to investigate the role of HPV-18 E6 and E6*I proteins in the Wnt/β-catenin signaling regulation. Through a TCF-4-dependent luciferase reporter plasmid, we show that E6 and E6*I up-regulate TCF-4 transcriptional activity, which is enhanced with the expression of exogenous β-catenin. Moreover, Wnt target genes are overexpressed in E6 and E6*I transfected cells. We also found that E6 and E6*I increase β-catenin and TCF-4 protein levels, but they do not alter their subcellular distribution. Immunoprecipitation and pull-down assays revealed an interaction of TCF-4 and β-catenin with E6 and E6*I proteins and those interactions impact in TCF-4 stabilization. We found that E6 and E6*I interact with the *Sp5* gene promoter, in vivo and in vitro. Furthermore, proliferation induced by β-catenin is enhanced by E6 and E6*I proteins. Finally, although E6 intratype variants differentially affected TCF-4 transcriptional activation, no differences appeared in their ability to bind TCF-4 or β-catenin.

## 2. Results

### 2.1. HPV-18 E6 and E6*I Proteins Enhance β-Catenin/TCF-4 Transcription

**Figure 1:**
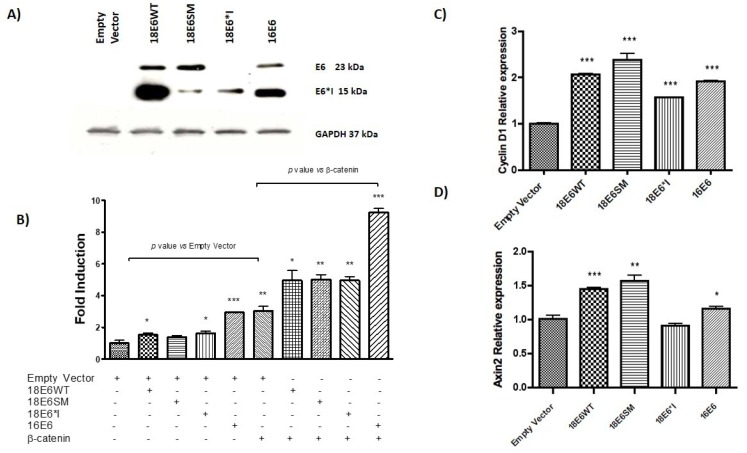
E6 and E6*I proteins induce TCF-4 transcriptional activity. (**A**) Expression of E6 and E6*I proteins was analyzed 48 h post-transfection in C33A cells, by western blot. (**B**) 18E6WT, 18E6SM, 18E6*I, 16E6, and β-catenin expressing vectors were transfected as indicated, with TOPFLASH (TCF-4 reporter plasmid) and β-galactosidase reporter plasmids in C33A cells. Luciferase reporter activity was measured 48 h post-transfection. Luciferase activities were compared with the empty vector or β-catenin plasmid. (**C**) *Cyclin D1* and (**D**) *Axin2* gene expression was evaluated by qPCR in E6 transfected cells. The means and ±SD of three independent experiments are depicted in each graph. Student t test was performed to evaluate the significant differences, the values are represented as * *p* < 0.05, ** *p* < 0.001, *** *p* < 0.0001.

C33A cells were transiently transfected with a TCF-4-dependent luciferase reporter plasmid (TOPFLASH) and FLAG-tagged versions of 18E6WT, 18E6SM, 18E6*I, or 16E6 expressing plasmids. All experiments were performed co-transfecting the empty vector (p3X) or the β-catenin expressing plasmid, as indicated. After 48 h of transfection, immunoblot assays were performed for each experiment, confirming the expression of FLAG-tagged E6 proteins (Figure 1A). It is worth mentioning that the relation of protein expression of E6 full length and E6*I in the 18E6WT transfected cells is around 20% and 80%, respectively; while in 18E6SM transfected cells, such a relation is inverted, being around 80% and 20%, respectively. This effect is because 18E6SM harbors an A233G mutation in the donor splicing site that promotes a decrease in the expression of E6*I. Therefore, 18E6SM was used to compare a condition with a higher expression of E6 full length. Ectopic expression of both 18E6WT and 18E6*I increased 1.5-fold TCF-4 transcriptional activity (Figure 1B), compared with the empty vector. 18E6SM showed a similar effect in the TCF4 transcriptional activation as observed for the other E6 expressing plasmids, although non-significant. A 2.9-fold induction of TCF-4 activity was observed in 16E6 transfected cells, similar to the effect of ectopically expressed β-catenin. Subsequently, when the Wnt pathway was over activated through the co-transfection of β-catenin and E6 expressing plasmids, an enhancement of TCF-4 activity occurred in all tested conditions, above the β-catenin response (around 1.6-fold). As shown in Figure 1B, the 18E6 full-length or E6*I continued showing an increase in TCF response, with a consistent higher effect for 16E6 (3-fold). 

Overexpression of E6 proteins also stimulate native promoters containing TCF-4 responsive elements, as evidenced by the expression of the Wnt target genes, *Axin2* and *Cyclin D1*, evaluated by qPCR assay. As observed in Figure 1C,D, E6 proteins enhanced the expression of *Cyclin D1* (up to 2-fold, compared to the control vector), while *Axin2* reached up to a 1.5-fold increase; although 18E6*I had no effect on *Axin2* expression. Taken together, these findings suggest that HPV-18 E6 and E6*I cooperate in the activation of the canonical Wnt/β-catenin cell signaling.

### 2.2. E6 Proteins Increase β-Catenin and TCF-4 Protein Levels, But Do Not Alter Their Subcellular Localization

**Figure 2:**
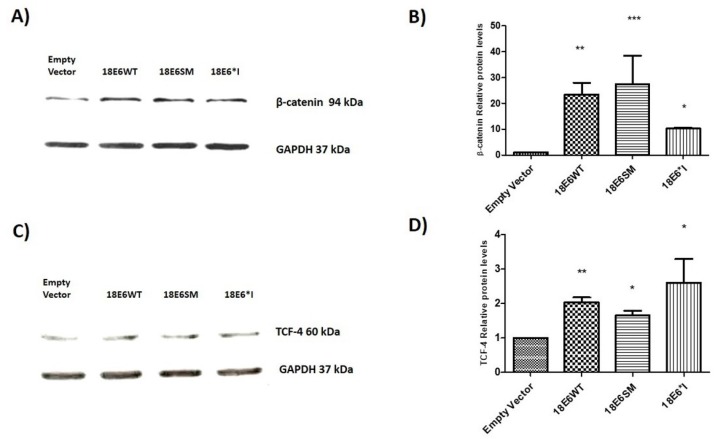
HPV-18 E6 and E6*I increase β-catenin and TCF-4 total protein levels. C33A cells were transfected with E6WT, E6SM, and E6*I expressing vectors. 48 h post-transfection, total cell lysates were analyzed by western blot. (**A**) β-catenin immunoblot and (**B**) densitometric analysis; (**C**) TCF-4 immunoblot; and (**D**) densitometric analysis. Data from three independent experiments were collected and graphed showing the mean and ±SD. *t* student analysis was performed, * *p* < 0.05, ** *p* < 0.001 and *** *p* < 0.0001 vs. empty vector values.

**Figure 3:**
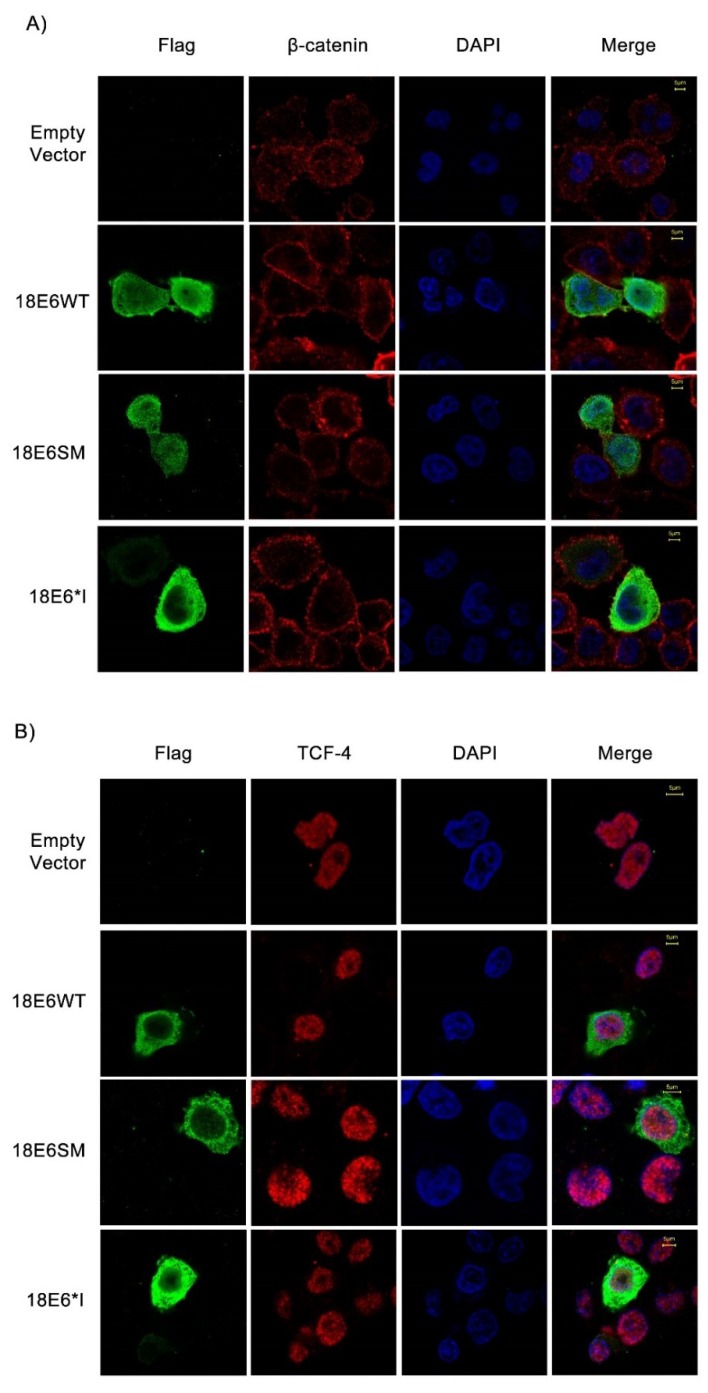
E6 proteins do not alter subcellular distribution of β-catenin or TCF-4. C33A cells were transfected with plasmids encoding 18E6WT, 18E6SM, or 18E6*I as indicated. 48 h post-transfection cells were fixed, and immunofluorescence stain was performed using specific antibodies against β-catenin (**A**) or TCF-4 (**B**) (Red) and FLAG (Green). Cells were also stained with DAPI (Blue) to visualize the nuclei. Images were acquired by confocal microscope. Data from three independent experiments were collected with a 63× objective oil immersion lens. Scale bar size 5 μm.

To determine the effect of E6 proteins on β-catenin and TCF-4 levels, we performed immunoblot assays using total cell lysates. We observed that both E6 and E6*I proteins significantly increase β-catenin (Figure 2A,B) and TCF-4 (Figure 2C,D) levels. Therefore, immunofluorescence assays were performed to investigate whether HPV-18 E6 full length and E6*I proteins alter β-catenin or TCF-4 localization. C33A cells were transfected with 18E6WT, 18E6SM, or 18E6*I expressing plasmids and after 48 h, cells were fixed and analyzed. As shown in Figure 3A,B, all the E6 proteins were detected in the cytosol and nuclei. On the other hand, β-catenin was found mainly at the cellular membrane and cytosol, which was unaffected by the presence of E6 proteins (Figure 3A). Similar results were obtained in HaCaT E6-transfected cells, a keratinocyte-derived model (Figure S1). Concordantly with previous reports carried out with 16E6, we observed that E6 and E6*I of HPV-18 do not alter β-catenin subcellular distribution [21]. As shown in Figure 3B, TCF-4 subcellular localization was also unaltered in the presence of the transfected E6 isoforms.

### 2.3. E6 Proteins Interact with the Wnt Activation Complex In Vivo and In Vitro

**Figure 4:**
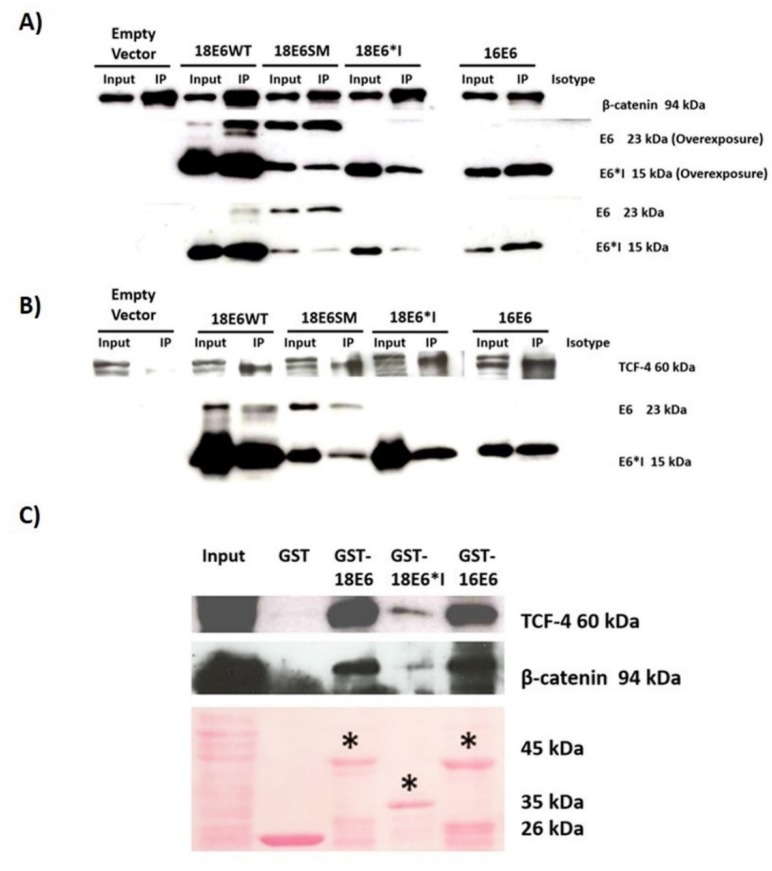
E6 and E6*I proteins interact with β-catenin and the transcriptional factor, TCF-4, in vivo and in vitro. C33A cells were transfected with different E6 expressing vectors, and 48 h post-transfection, protein lysates were obtained. (**A**) β-catenin and (**B**) TCF-4 were immunoprecipitated with the appropriate antibodies. The immuno-complexes were analyzed by Western blot using anti-β-catenin and anti-TCF-4 antibodies to detect the immunoprecipitated protein, and with an anti-FLAG to detect E6 proteins. Image shows a representative experiment of three performed. For comparison, 10% of protein used for immunoprecipitation (input) and the precipitation with an irrelevant IgG antibody (isotype) are shown. An overexposure of E6 proteins in shown in panel A. (**C**) Purified GST-18E6, GST-18E6*I, and GST-16E6 recombinant proteins were incubated with C33A protein extracts, while GST purified protein was used as a control. Immunoblots were performed using anti-β-catenin and anti-TCF-4 antibodies. 10% of protein extract was used as input. Lower panel shows Ponceau S red staining of a representative nitrocellulose membrane. Asterisks (*) show the E6 recombinant proteins.

Previous reports demonstrate that HPV-16 E6 interacts with members of the Wnt signaling pathway, such as Dvl2 (Dishevelled Segment Polarity Protein 2) [23]. To investigate a further interaction with β-catenin and TCF-4, immunoprecipitation, assays were performed in C33A cells transfected with the different E6 expressing plasmids. After 48 h post-transfection, cell protein lysates were obtained and incubated with anti-β-catenin or anti-TCF-4 specific antibodies to immunoprecipitate these proteins. Afterwards, a western blot with anti-FLAG antibody was performed to assess the binding of the E6 proteins with β-catenin or TCF-4. As observed in Figure 4A,B, the immunoblot revealed that 16E6, 18E6 full-length, and 18E6*I proteins were able to bind to β-catenin (94 kDa band) and TCF-4 (60 kDa band), respectively. 

These results were further confirmed by GST pull down assays using C33A lysates and GST-E6 fusion proteins (Figure 4C), where recombinant proteins bound to β-catenin and TCF-4. These data suggest that the E6 proteins regulate the Wnt/β-catenin cell signaling pathway through the interaction with the TCF-4 activation complex.

### 2.4. E6 and E6*I from HPV-18 Increase TCF-4 Protein Stability

**Figure 5:**
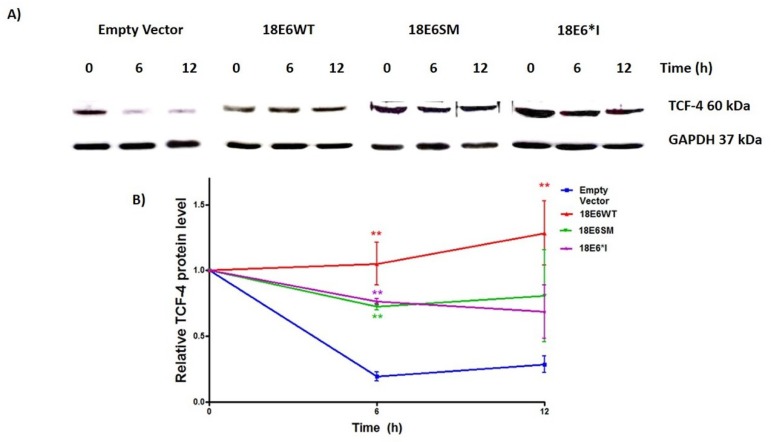
HPV-18 E6 proteins augment TCF-4 stability. C33A cells were transfected with E6 expressing plasmids. 48 h post-transfection, 200 µg/mL of cycloheximide was added to the culture medium. Protein extracts were obtained at 0, 6, and 12 h after treatment. (**A**) A representative immunoblot is shown with the different treatments. In non-E6 transfected cells, the TCF-4 levels were diminished at 6 and 12 h post-treatment, in contrast to E6 expressing cells, where TCF-4 levels remained without change at 6 and 12 h. (**B**) Graph showing the data as the mean and ± SD of three independent experiments. One-way ANOVA and a Tukey’s post-hoc test, ** *p* < 0.001 versus empty vector values.

To further determine the effect of the interaction of HPV-18 E6 and E6*I with TCF-4, we evaluated the TCF-4 stability through half-life determination assay. C33A cells were transfected with E6 or E6*I expressing plasmids, and 48 h post-transfection, cells were treated with 200 µg/mL of cycloheximide and the TCF-4 degradation rate was evaluated at 0, 6, and 12 h post-treatment. Overtime, it was observed that TCF-4 protein levels decreased considerably in E6 non-transfected cells after 6 and 12 h (Figure 5A,B). Interestingly, TCF-4 protein levels were maintained in the presence of E6 and E6*I proteins after 6 h, reaching up to 4.37- to 7.25-fold, compared to cells transfected with the empty vector. Finally, TCF-4 protein levels were higher at 12 h in 18E6WT expressing cells compared to those transfected with the control vector. These data strongly suggest that the half-life of TCF-4 is elongated in E6 and E6*I expressing cells, and that such an effect could be explained through the E6/E6*I-TCF-4 interaction.

### 2.5. HPV-18 E6 and E6*I Increase Nuclear TCF-4 Protein Levels 

**Figure 6:**
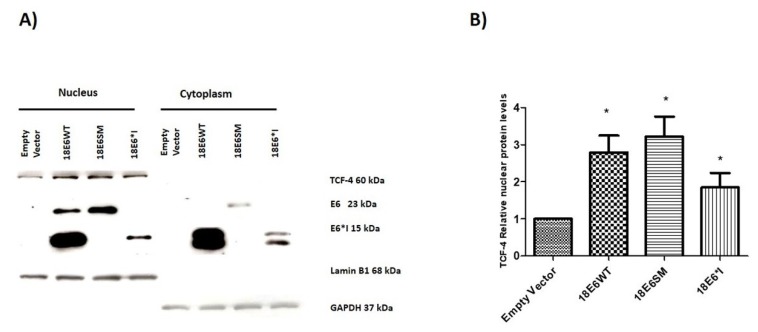
E6 proteins increase nuclear TCF-4 protein levels. (**A**) Representative immunoblot of TCF-4 and E6 proteins in nuclear and cytoplasmic soluble fractions of C33A cells transfected with E6 expressing plasmids. Lamin BI and GAPDH proteins were used as nuclear and cytoplasmic load controls, respectively. (**B**) A densitometric analysis of relative nuclear TCF-4 levels shows an increase of TCF-4 protein levels in the presence of the E6 proteins. Data from three independent experiments were collected and graphed showing the mean and ± SD. *t* student analysis was performed, * *p* < 0.05 vs. empty vector values.

To further determine the effect of E6 proteins on nuclear TCF-4 levels, soluble cellular fractionation was performed. As is shown in Figure 6A, full-length E6 is mainly located in the nucleus while E6*I is found in both the nucleus and cytoplasm. Interestingly, TCF-4 was significantly increased in the nucleus in both E6 and E6*I expressing cells (Figure 6A,B). This supports our data showing an increase in TCF-4 stability, which may lead to an enrichment of nuclear TCF-4.

### 2.6. HPV-18 E6 and E6*I Proteins Bins to a TCF-4 Dependent Promoter In Vivo and In Vitro

**Figure 7:**
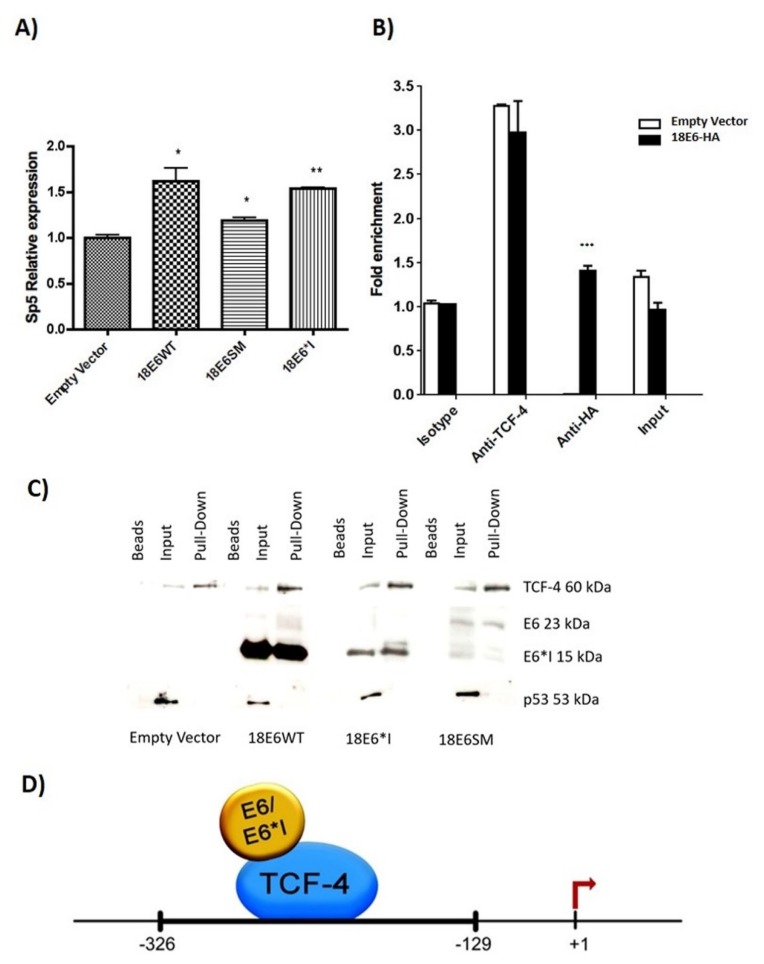
E6 and E6*I of HPV-18 interact with the *Sp5* promoter. C33A cells were co-transfected with β-catenin and E6 expressing plasmids as indicated: (**A**) E6 proteins increase *Sp5* relative expression as shown by qPCR analysis; * *p* < 0.05, ** *p* < 0.01, compared to the empty vector; (**B**) Chromatin immunoprecipitation assay (ChIP) shows that 18E6 binds to the *Sp5* promoter in vivo. Anti-HA antibody was used to detect E6-HA tagged protein, and anti-TCF-4 and anti-IgG antibodies were used as positive and isotype controls, respectively. 10% of input was analyzed. *** *p* < 0.001, of E6-HA compared to the empty vector. (**C**) C33A cells were transfected with 18E6WT, 18E6SM, or 18E6*I expressing plasmids, and 48 h post-transfection, a DNA pull-down assay was performed. As expected, in all the samples, TCF-4 interact with the *Sp5* promoter probe in vitro. Interestingly, both E6 full-length and E6*I form a complex with the *Sp5* promoter. The p53 transcriptional factor was used as a negative control since it does not bind to the *Sp5* promoter. (**D**) Scheme showing the suggested interactions of E6 and E6*I of HPV-18 with the TCF-4 dependent promoter, proposing a possible mechanism of Wnt cell signaling regulation by E6 proteins.

The effect of E6 proteins in *Sp5* expression was analyzed by qPCR. Figure 7A demonstrates that E6 proteins increase *Sp5* mRNA levels in C33A cells co-transfected with β-catenin. Therefore, we further analyzed whether E6 proteins could interact with the *Sp5* promoter, which is TCF-4-dependent. It was previously demonstrated that TCF-4 binds to a conserved sequence, A-C/G-A/T-T-C-A-A-A-G, found in TCF-4/β-catenin-dependent promoters, such as the *Sp5* gene promoter [24,25]. We used a pair of primers to amplify a *Sp5* promoter sequence flanking the -326 to -129 nucleotides, since this region contains two TCF-4 binding sites located at nucleotides, -303 to -296, and -149 to -142. Those binding sites were confirmed when the fragment was analyzed in silico using the informatics tool, Tfsitescan [26]. Co-transfections of E6-HA-tagged and β-catenin plasmids were performed in C33A cells. Chromatin immunoprecipitation assay (ChIP) revealed that 18E6 binds to the *Sp5* promoter (Figure 7B). TCF-4 also bound to this promoter, either in cells with the empty vector or 18E6-HA transfected cells. It is worth mentioning that the binding of 18E6 to the *Sp5* promoter is overwhelming, since virtually no amplification is seen when the immunoprecipitation is carried out with anti-HA in cells transfected with the empty vector. 

In order to confirm the obtained results, C33A cells were transfected with 18E6WT, 18E6SM, or 18E6*I expressing plasmids, and 48 h post-transfection, the DNA pull-down assay was performed. As expected, TCF-4 interacted with the *Sp5* promoter in all the tested samples (Figure 7C). Interestingly, E6 full-length as well as E6*I were also able to interact with the *Sp5* promoter. As a negative control, we used the p53 transcription factor, which has no response elements within the *Sp5* promoter. It is worth mentioning that even though p53 binds to E6 and E6*I, p53 was not found forming a complex in the *Sp5* fragment (Figure 7D). Taken together these results indicate indicates that E6 and E6*I proteins from HPV-18 interact with a TCF-4 dependent promoter, suggesting that such binding could be performed through TCF-4/E6/E6*I interactions, which may allow the up-regulation of the Wnt/β-catenin signaling pathway (Figure 7D).

### 2.7. HPV-18 E6 and E6*I Proteins Induce Cell Proliferation in Cooperation with β-Catenin Overexpression

**Figure 8:**
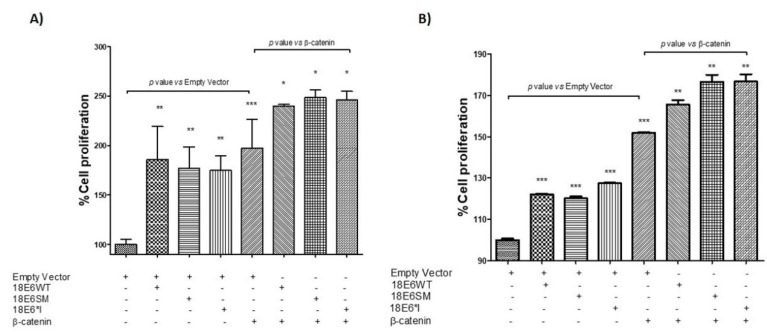
HPV-18 E6 and E6*I alone or in combination with β-catenin increase cell proliferation. C33A cells were transfected with the indicated plasmids, and 24 h post-transfection were seeded into a 96 well plate. Then, experiments were assessed after 72 h either by (**A**) MTS (3-(4,5-dimethylthiazol-2-yl)-5-(3-carboxymethoxyphenyl)-2-(4-sulfophenyl)-2H-tetrazolium, inner salt) or (**B**) Crystal violet assays. Data from three independent experiments were collected and graphed showing the mean and ± SD. *t* student analysis was performed, * *p* < 0.05, ** *p* < 0.001 and *** *p* < 0.0001 vs. empty vector values.

Finally, to determine the contribution of E6 proteins in the Wnt/β catenin signaling pathway, MTS and crystal violet proliferation assays were performed in C33A cells co-transfected with β-catenin. As shown in Figure 8A,B, when E6 proteins were transfected alone, there was an increase in proliferation of between 70 and 85% in MTS assays, while crystal violet assays showed an increase of only 20–30% in relation to the empty vector. Additionally, transfection of β-catenin alone showed an increase in proliferation of 97% in MTS assays and 51% in crystal violet assays. Furthermore, when both E6 and E6*I and β-catenin were co-transfected, there was a further increase in proliferation of 40–50% in MTS assays, as compared to β-catenin alone, while the increase found using crystal violet was of 15-30%. This suggests that E6 proteins cooperate with β-catenin to promote the proliferation of these cells.

### 2.8. HPV-18 E6 Variants Differentially Modulate TCF-4-Mediated Transcription 

**Figure 9:**
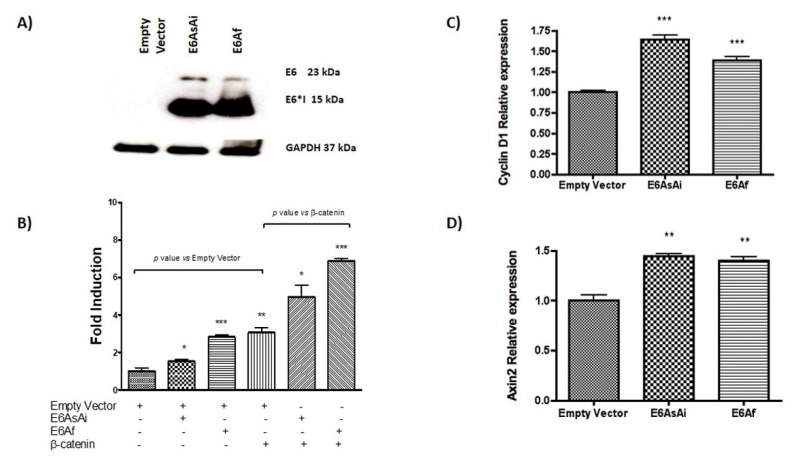
HPV-18 E6AsAi and E6Af variants modulate TCF4 transcriptional activity. (**A**) HPV-18 E6AsAi and E6Af protein expression in C33A transfected cells. (**B**) C33A cells were transfected with E6AsAi, E6Af, alone or combined with β-catenin expressing plasmids, and co-transfected with TCF-4 transcriptional reporter plasmid (TOPFLASH) and β-galactosidase reporter vector as indicated. (**C**) *Cyclin D1* and (**D**) *Axin2* gene expression was analyzed by qPCR. The means and ±SD of three independent experiments are depicted in each graph. Student t test was performed to evaluate the significant differences, the values are represented as * *p* < 0.05, ** *p* < 0.001, *** *p* < 0.0001.

It has been proposed that HPV variants of the same type may present distinct biological behaviors conferring different pathogenic risks [17,27,28]. To determine whether HPV-18 E6 variations differentially induce TCF-4 transcriptional activity, we tested the E6Af variant belonging to the African phylogenetic branch and harbors genomic variations that lead to amino acidic changes, compared to the reference variant, E6AsAi, that in this study, is also shown as 18E6WT [17]. HPV-18 E6Af and E6AsAi expressing plasmids were transfected in C33A cells and co-transfected with the TCF-4-dependent luciferase reporter plasmid (TOPFLASH), β-galactosidase reporter, and, in some cases, with β-catenin plasmids, as indicated. Protein expression of E6 variants was evaluated by immunoblot as shown in Figure 9A. E6Af was able to augment TCF-4 transcriptional activity up to 2.8-fold, higher than the 1.5-fold induction observed for E6AsAi. These effects were also evident when β-catenin was added, where E6Af reached up to 2.25-fold induction, while E6AsAi showed a 1.6-fold induction above exogenous β-catenin. These results show that HPV-18 E6 variants differentially induce TCF-4 transcriptional activation (Figure 9B). However, when the levels of Wnt target native genes were analyzed by qPCR, both E6 variants were able to enhance the expression of *Axin2* and *Cyclin D1*, with no significant differences among them, as shown in (Figure 9C,D).

### 2.9. HPV-18 Intratype Variants Interact with β-Catenin and TCF-4

**Figure 10:**
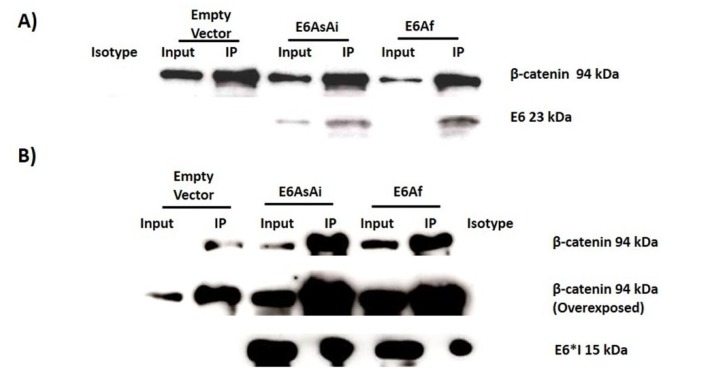

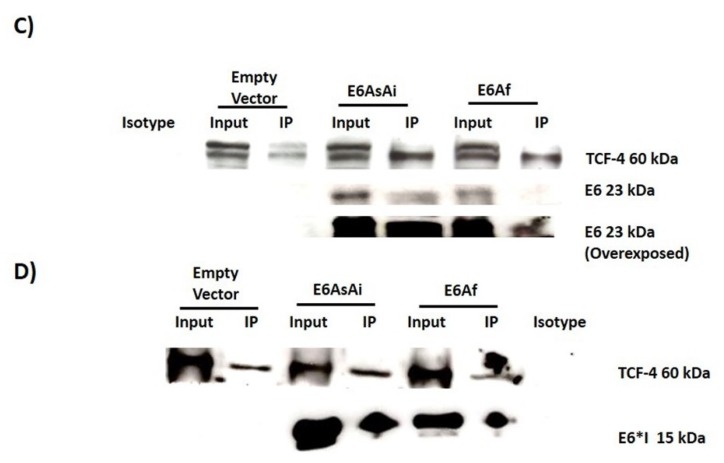
HPV-18 E6 variants interact in vivo with β-catenin and TCF-4. C33A cells were transfected with the E6 variant plasmids. 48 h post-transfection, cell lysates were collected and immunoprecipitated with anti-β-catenin (**A**,**B**) and anti-TCF-4 (**C**,**D**). Immunoblots show an interaction of E6 (**A**,**C**) and E6*I (**B**,**D**) with both proteins. Overexposures of β-catenin and E6 proteins are shown in panel B and C, respectively. Representative images are shown from three experiments performed. 10% of protein used for immunoprecipitation is indicated as input, and an irrelevant antibody was used (isotype).

In order to demonstrate the interaction of β-catenin and TCF-4 proteins with E6AsAi or E6Af variants, immunoprecipitation assays were done. C33A cells were transfected with E6AsAi and E6Af expressing plasmids, and 48 h post-transfection cells were lysed and incubated with anti-TCF-4 and anti-β-catenin specific antibodies. Immunoblot analysis revealed that both E6 variants were able to interact with both β-catenin (Figure 10A,B) and TCF-4 (Figure 10C,D), respectively. Therefore, although the tested E6 variants showed a differential effect in other cellular pathways [29], no changes were observed in the ability to bind TCF-4/β-catenin nor in the levels of the regulated genes.

## 3. Discussion

The continuous expression of the E6 and E7 oncoproteins is necessary for cellular transformation and immortalization in HPV-related cancers. E6 oncoprotein contributes to malignant progression through targeting a set of cellular proteins [30]. A common feature of E6-E7 mRNA from HR-HPV is to produce small isoforms termed E6*, whose cellular functions are poorly understood [18]. It is proposed that HPV-18 E6*I antagonizes the effects of full-length E6 [31], while some studies show that E6* has proper E6-independent functions [32,33,34,35]. E6* interacts with E6 and E6AP [31], and also downregulates PDZ domain containing proteins, such as hDlg, Scrib, MAGI-1, and MAGI-2 [32]. It has also been demonstrated that E6* modulates apoptosis related-proteins, since it binds to procaspase 8, affecting its stability [36]. Our research group previously showed that E6* induces the activation and nuclear translocation of the procaspase 8, without inducing cell death [33]. These findings suggest that E6* isoforms may, in a way that has not yet been described, cooperate with E6 in the malignant progression.

An aberrant activation of the Wnt cell signaling pathway has been described during cervical carcinogenesis [9,20,37] and a role of HPV-16 E6 in this activation has been proposed in cellular models [8,21,22,38]. Nevertheless, the effect of HPV-18 E6 and E6*I in this pathway has not yet been analyzed.

In the present study, we demonstrate that E6*I hyperactivates the Wnt/β-catenin pathway. HPV-18 E6*I by itself enhanced the TCF-4 transcriptional activity in C33A-transfected cells in 1.5-fold compared to the control, and a similar effect was observed when 18E6WT was expressed. This suggests that E6*I cooperates with E6 full length in the TCF-4 response. In an attempt to increase the expression of E6 over E6*I, we used an E6 expressing plasmid whose expression is enriched with full-length E6 (18E6SM). The mutation in the 18E6SM plasmid allows the expression of higher amounts of E6 full-length, although some E6*I is still produced since another donor site not yet described could be used during the splicing process. When transfecting this plasmid there was a slight increment in the TCF-4 transcriptional regulation, although non-significant, in relation to the control vector. 

Interestingly, when the Wnt pathway was over activated with the co-transfection of β-catenin, TCF-4 transcriptional activation was enhanced in the presence of E6 or E6*I proteins, supporting that E6*I and E6 collaborate in Wnt/β-catenin pathway activation. We also found that 16E6 induced a higher response in TCF-4 activation, reaching up to a 2.5-fold induction, and this effect was slightly enhanced in the presence of exogenous β-catenin (3-fold). Our results are in agreement with previous reports, where three-fold-induction of the TCF-4 luciferase reporter was obtained in HEK293T cells ectopically expressing HPV-16 E6, the Wnt receptor HFz1, and the Wnt3a ligand [21]. Additionally, Bonilla-Delgado et al. (2012) [23] reported a 50-fold induction of the TCF-4-dependent response in COS-7 cells where HPV-16 E6 in combination with Dvl-2 and β-catenin were expressed. These data suggest that the cellular context and/or the method used for Wnt pathway activation play an important role in the effect induced by HPV E6. 

The expression of TCF-4 target genes was evaluated in the presence of E6 and E6*I. In HPV-18, E6WT and E6SM expressing cells increased *Cyclin D1*, *Axin2*, and *Sp5* expression, in agreement with a previous report where E6 from HPV-16 was able to activate the Cyclin D1 promoter [21]. Moreover, in a transgenic mice model expressing HPV-16 E6, *Cyclin D1* was also up-regulated [23].

Among the Wnt target genes, *Axin2* is considered a tumor suppressor and mutations within this gene are associated to cancer development [39,40]. Axin2 is involved in a Wnt negative feedback loop that may limit the duration intensity or the spread of Wnt signaling [41,42]. It is interesting that even though E6*I increased *Cyclin D1* expression, it failed in promoting an up-regulation of *Axin2*. It is possible that E6 recruits co-regulators that are distinct to those recruited by E6*I that could be necessary for *Axin2* expression.

It has been previously demonstrated that HPV-16 E6 induces TCF-4 transcriptional activation without affecting β-catenin localization [21]. Consistent with this result, our findings revealed that although E6 and E6*I increase β-catenin and TCF-4 protein levels, they do not alter their subcellular localization. Moreover, our results demonstrate that both E6 and E6*I are able to complex with β-catenin and TCF-4 in vivo and in vitro, which are the main proteins involved in the TCF-4-dependent transcriptional activation. 

We demonstrate that E6 and E6*I from HPV-18 not only interact with TCF-4, but are also able to induce the TCF-4 stabilization. Further studies are needed to elucidate whether the interaction of TCF-4 with E6 proteins is responsible for TCF-4 stabilization. Previous studies have shown that HPV-16 E6 together with the E3 ubiquitin ligase E6AP, and induce stabilization of other members of the canonical Wnt pathway, such as β-catenin, impacting in the activation of the pathway [22]. 

Furthermore, it is well known that when Wnt signaling is activated, β-catenin complexes with TCF-4 in the nucleus, inducing the TCF-4 transcriptional response [43]. Our findings revealed that both E6 and E6*I increased nuclear TCF-4 protein levels, which may directly impact in TCF-4 transcriptional activation. Remarkably, the DNA pull-down as well as the ChIP results revealed that both E6 and E6*I interact with a TCF-4 dependent promoter. This interaction could be explained through complexes formed by E6/E6*I and TCF-4 that recognize specific sequences located at the *Sp5* promoter or other TCF-4 response promoters. Additionally, in concordance with previous studies [44,45], we found an enhancement in proliferation in E6 expressing cells. Interestingly, proliferation enhanced by β-catenin was increased when E6 and E6*I were co-transfected. Therefore, our findings support a new mechanism by which E6 and also E6*I could modulate the Wnt/β-catenin pathway. 

Since HPV intratype variations have been proposed to affect the HPV biological behavior, we were also interested in determining if HPV-18 E6 variants could differentially affect the Wnt/β-catenin signaling pathway. HPV intratype variants are defined as those containing less than 1% of nucleotide changes in coding regions [46,47]. Our group has previously reported that HPV-18 variants exhibit differences in E6 full-length/E6*I transcript proportions, impacting on p53 levels [17]. Moreover, E6 variants differentially modulate the Akt/PI3K signaling pathway [29]. Previous studies demonstrated that E6 variants from HPV-16 exert different abilities in the activation of the Wnt/β-catenin signaling pathway [48]. Luciferase assays described herein showed that HPV-18 E6 variants have a different ability to augment TCF-4 dependent transcription, showing that E6Af promoted at least a 2.8-fold induction of the reporter gene transcription compared with 1.5-fold of E6AsAi. In addition, when E6 variants where co-transfected with β-catenin, E6-Af and E6AsAi reached up to a 2.25-fold and a 1.6- fold TCF-4 induction, respectively, compared to that obtained with β-catenin exogenous expression. Nevertheless, even though E6 variants displayed different levels of TCF-4 activation, both E6Af and E6AsAi enhanced the expression of *Axin2* and *Cyclin D1*, with no differences among them. This effect could be due to a distinct capacity of E6 variants to regulate or interact with other untested proteins involved in the activation of the Wnt signaling pathway. However, we observed that both E6 variants interact with TCF-4 and β-catenin in a similar manner, which reveals that aminoacidic changes in E6 variants do not influence at least in such a binding capacity.

In this study, we demonstrated that not only E6, but also E6*I from HPV-18 are able to up-regulate Wnt/β-catenin signaling, involving their interaction with the TCF-4 activation complex. Additional effects on members of the Wnt pathway should be analyzed in order to determine the specific contribution of E6 and the spliced isoform E6*I in cell transformation induced by the Wnt/β-catenin pathway.

## 4. Materials and Methods

### 4.1. Cell Culture and Transfection

C33A epithelial cells were acquired from ATCC and HaCaT were kindly provided by A. García-Carrancá (Instituto Nacional de Cancerología, Mexico City, Mexico) and were maintained in Dulbecco’s modified Eagle medium (DMEM) supplemented with 10% of fetal bovine serum (FBS) in a humidified incubator with 5% CO_2_. Transfections were performed using Lipofectamine 2000 reagent (Invitrogen, Carlsbad, CA, USA) according to the manufacturer’s instructions.

### 4.2. Plasmids

18E6WT Open Reading Frame (ORF) was obtained and PCR-amplified from an HPV-18 positive cervical cancer biopsy, 18E6*I ORF was obtained by RT-PCR amplification from HeLa cells (HPV-18 positive), and 16E6 ORF was amplified by PCR from CaSki cells (HPV-16 positive). The HPV-18 E6 spliced mutant (18E6SM) sequence was amplified from the plasmid, pCAHPV18-E6sm [32]. 18E6SM harbors a mutation at the donor splicing site (G233A), favoring the expression of the E6 full-length. The HPV-18 E6Af variant (from African phylogenetic branch) and the E6AsAi variant (from Asian-Amerindian phylogenetic branch), which is the canonical reference variant, were PCR-amplified from DNA previously obtained from tumor biopsies [27]. All these fragments were purified and cloned into the p3x-FLAG CMV.10 expression vector (Sigma Aldrich, Sant Louis, MO, USA) Constructs were verified by DNA-sequencing. β-catenin, pCAHPV18-E6sm, and pGW1-18E6-HA (hemagglutinin-tagged) expressing plasmids were kindly provided by Lawrence Banks (ICGEB, Trieste, Italy). The TCF-4 reporter plasmid (TOPFLASH) containing two sets of 3 copies of TCF-4 binding sites upstream of Thymidine Kinase minimal promoter and luciferase ORF (Merck-Millipore, Burlington, MA, USA) was used to perform luciferase assays, and pCMV-β-galactosidase plasmid (Promega, Madison, WI, USA) was used to evaluate the efficiency of transfection.

### 4.3. Luciferase Reporter Activity Assays

C33A cells were seeded in a 24 well plate and transfected with a mix containing 50 ng of appropriate E6 expressing plasmid, 100 ng of TOPFLASH, and 1 ng of β-galactosidase reporter plasmid, either with 50 ng of empty vector or β-catenin, as indicated. Cell extracts were obtained 48 h post-transfection and assayed for luciferase and β-galactosidase activities (Tropix Inc, Bedford, MA, USA) using a Glomax 96-well plate luminometer (Promega, Madison, WI, USA) The presented data are shown as relative luciferase readouts comparing the E6 expressing cells vs the control vector, where luciferase readouts in empty vector condition were adjusted to 1 after normalizing with β-galactosidase activity. At least three independent experiments were performed, each by triplicate.

### 4.4. Quantitative Polymerase Chain Reaction (qPCR)

C33A cells were seeded in a 60 mm culture dish and transfected with 3 µg of each E6 plasmid. After 48 h post-transfection, cells were collected, and total RNA extraction was performed using the RNeasy mini kit (Qiagen, Hilden, Germany). The isolated RNA was treated with the DNAse Free DNA removal kit (Thermo Fisher Scientific, Waltham, MA, USA) and 400 µg of RNA was reverse-transcribed with random hexamers utilizing the GeneAmp RNA PCR Core Kit (Applied Biosystems, Foster City, CA, USA) For the *Cyclin D1* amplification, forward 5′-ACAAACAGATCATCCGCAAACAC-3′ and reverse 5′-TGTTGGGGCTCCTCAGGTTC-3′ primers were used. For *Sp5* amplification, forward 5’-TCGGACATAGGGACCCAGTT-3′ and reverse 5′-CTGACGGTGGGAACGGTTTA-3′. As a house keeping control, 18S mRNA was amplified with forward 5’-AACCCGTTGAACCCATT-3′ and reverse 5’-CCATCCAATCGGTAGTAGCG-3′ primers. SYBER select Master Mix (Applied Biosystems, Foster City, CA, USA) was utilized for qPCR reactions. For Axin2 amplification, Taqman probes were used (Applied Biosystems, Foster City, CA, USA): *Axin2* FAM (Hs00610344_m1) and 18S VIC (Hs99999901_s1) probes, with Taqman Gene Expression Master Mix for qPCR analysis (Applied Biosystems, Foster City, CA, USA). The results are presented as relative quantification using the ^ΔΔ*C*t^ method.

### 4.5. Western Blotting

C33A cells were cultured in 60 mm dishes and transfected with 3 µg of the indicated plasmid. 48 h post-transfection, cells were lysed using 300 µL of RIPA buffer (100 mM Tris pH 8.0, 50 mM NaCl_2_, 0.5% Nonidet P-40, and protease inhibitor cocktail (Roche, Basel, Switzerland)). 20 μg of cell protein extracts were analyzed by SDS-PAGE gels (10–12%) and transferred in a 0.22 µm nitrocellulose membrane (Bio-Rad). Membranes were blocked with 10% skimmed milk in TBS-0.1% Tween 20 per 1 h at room temperature, followed by incubation with the indicated primary antibody diluted 1:1000: anti-FLAG M2 (Sigma Aldrich, Sant Louis, MO, USA); anti-TCF-4 (Santa Cruz Biotechnologies, Dallas, TX, USA); anti-β-catenin (Santa Cruz Biotechnologies, Dallas, TX, USA). After washing three times with TBS-0.1% Tween 20, membranes were incubated with HPR-conjugated secondary anti-mouse antibody in a dilution 1:10000 (Santa Cruz, Biotechnologies, Dallas, TX, USA). Proteins were visualized utilizing the Immobilon Western (Millipore) according to the manufacturer’s instructions. Western blots were performed at least three times each to assure result reproducibility.

### 4.6. Immunoprecipitation Assay

After 48 h of transfection with the indicated plasmid, 400 µg of protein extracts were incubated with 1µg of anti-β-catenin (Santa Cruz Biotechnologies, Dallas, TX, USA), anti-TCF-4 (Santa Cruz Biotechnologies, Dallas, TX, USA) antibodies, or IgG isotype control (Santa Cruz Biotechnologies, Dallas, TX, USA) overnight at 4 °C. A total of 20 µL of protein G-agarose beads (Upstate) were added to each sample and incubated at 4 °C, for 3 h. Complexes were washed three times with PBS-0.1% NP-40, resuspended in Laemmli sample buffer, and submitted to immunoblot analysis with anti-FLAG M2 (Sigma Aldrich, Sant Louis, MO, USA), anti-β-catenin (Santa Cruz Biotechnologies, Dallas, TX, USA), and anti-TCF-4 antibodies (Santa Cruz Biotechnologies, Dallas, TX, USA).

### 4.7. Analysis of TCF-4 Stability

C33A cells were seeded in 60 mm dishes and transfected with 3 µg of the indicated plasmid. 48 h post-transfection, cells were treated with 200 µg/mL of cycloheximide (an inhibitor of protein biosynthesis) (Sigma Aldrich, Sant Louis, MO, USA). After 0, 6, and 12 h post-treatment protein extracts were isolated using 2× Laemmli sample buffer (Bio-Rad, Hercules, CA, USA). Western blot assays were carried out in order to analyze the TCF-4 protein stability.

### 4.8. Immunofluorescence Staining and Cell Imaging

C33A and HaCaT cells were seeded over slides in 6 well plates and transfected with the indicated plasmids. After 48 h post-transfection cells were fixed with 3.7% paraformaldehyde in PBS for 10 min and permeabilized with PBS-0.1% Triton X-100. Then, cells were incubated with anti-FLAG M2 (Sigma Aldrich, Sant Louis, MO, USA) and anti-β-catenin (Cell Signaling) or anti-TCF-4 (Santa Cruz Biotechnologies, Dallas, TX, USA) antibodies overnight at 4 °C, after blocking with a 0.3% BSA solution. Cells were washed extensively with PBS and later incubated with anti-rabbit or anti-mouse antibodies conjugated to Rhodamine or Alexa-488 (Invitrogen, Carlsbad, CA, USA), respectively. Slides were washed and mounted with Prolong Diamond Antifade Mounting (Molecular Probes, Eugene, OR, USA) and then analyzed with a confocal microscope (Zeiss LSM 710 DUO, Oberkochen, Germany), with lasers giving excitation lines at 488 and 594 nm. Around twenty fields were observed for each treatment and representative images were acquired. The data of three independent experiments were collected with a 63× objective oil immersion lens.

### 4.9. GST- Fusion Protein Purification

E6 coding sequences were cloned into the pGEX-2T (GE) expression plasmid and the identity of each plasmid was verified by DNA-sequencing. GST-fusion protein production was induced in DH5-α E. coli strain with 10 mM IPTG. After three hours of induction, proteins were purified by lysing the cells using 1% triton/PBS and separating the insoluble fraction by centrifugation. Supernatant was then incubated with glutathione sepharose beads (Sigma Aldrich, Sant Louis, MO, USA), washed several times, and then re-suspended in 1 mL of 1% triton/PBS and analyzed by SDS-PAGE. Similar amounts of GST-fusion proteins were incubated overnight with 40 µg of C33A cellular protein extract, beads were then washed several times, and bound protein was analyzed by western blot using anti-TCF-4 and β-catenin antibodies.

### 4.10. Soluble Cell Fractionation Assay

C33A cells were seeded into a 60 mm dish and transfected with 3 µg of the indicated plasmid. 48 h post-transfection, cells were pelleted and washed with PBS (Phosphate-Buffered Saline). Cells were resuspended in 300 µL of lysis buffer (10 mm Tris pH 6.5, 27 mM Na_2_S_2_O_5_, 1% Triton X-100, 10 mM MgCl_2_, 25 mM Sucrose, and protease inhibitor cocktail) and incubated for 10 min at 4 °C with gentle agitation. The samples were centrifuged and the supernatants were collected (Cytoplasmic fraction). The pellets were resuspended in extraction buffer (10 mM HEPES pH 7.9, 10 mM KCl, 0.1 mM EDTA pH 8.0, 0.1 mM EGTA pH 8.0, and protease inhibitor cocktail) and centrifuged through a 0.34 M sucrose gradient. Then, the pellets were resuspended in RIPA buffer (100 mM Tris pH 8.0, 50 mM NaCl_2_, 0.5% Nonidet P-40, and protease inhibitor cocktail (Roche, Basel, Switzerland)) (Nuclear fraction). The samples were analysed by immunoblot using anti-TCF-4 (Santa Cruz Biotechnologies, Dallas, TX, USA), anti-Lamin B1 (Abcam, Cambridge, UK), anti-GAPDH (Santa Cruz Biotechnologies, Dallas, TX, USA), and anti-FLAG M2 (Sigma Aldrich, Sant Louis, MO, USA) antibodies.

### 4.11. DNA Pull-Down Assay

A fragment of the *Sp5* promoter was amplified using the biotin labelled forward primer, 5′Bio-GGGTCTCCAGGCGGCAAG3′, and reverse specific primer, 5′-AGCGAAAGCAAATCCTTTGAATCC-3′. The probe was purified with the QIAquick PCR purification kit (Qiagen, Hilden, Germany) according to the manufacturer’s protocol. C33A cells were seeded and transfected with 10 μg of each plasmid as indicated. 48 h post-transfection cells were lysed using the HKMG buffer (10mM HEPES pH 7.9, 100 mM KCl, 5 mM MgCl_2_,1 mM DTT, 1 mM Na_3_VO_4_, 10% glycerol, 0.5% NP-40, and protease inhibitor cocktail), incubated 20 min at 4 °C, and then passed through a 25-gauge needle attached to a 1 mL syringe for 20 times. The lysates were centrifuged, and the supernatant were collected. The protein extracts were pre-cleared with 60 μL of Streptavidin agarose beads (Invitrogen, Carlsbad, CA, USA) during 30 min at 4 °C and then were centrifuged and the supernatants were collected. For each sample, a total amount of 4 µg of biotin probes and 2.5 µg of Poly dI-dC (Sigma Aldrich, Sant Louis, MO, USA) were added and incubated overnight. 60 µL of Streptavidin agarose beads were added to each sample and incubated during 30 min at 4 °C. The samples were centrifuged, and the supernatants were discarded. The obtained beads were washed five times with the HKMG buffer and finally resuspended with 2× Laemmli sample buffer (Bio-Rad, Hercules, CA, USA). Then, Western blot assays were carried out.

### 4.12. Chromatin Immunoprecipitation Assay

C33A cells were seeded in a 100 mm plates and co-transfected with 7 µg of each 18E6HA and β-catenin expressing plasmids. 48 h post-transfection, cells were cross-linked with 1% of formaldehyde and quenched with 0.125 M of glycine. Cell lysates were obtained using a Lysis Buffer (1% SDS, 10 mM EDTA pH 8, 50 mM Tris HCl pH 8, and protease inhibitor cocktail) and sonicated with a Bioruptor Pico (Diagenode, Denville, NJ, USA), obtaining DNA fragments ranging from 200 to 500 bp. A total of 20 µg of chromatin per sample was used and diluted 1:5 with a Dilution Buffer (1% Triton X-100, 150 mM NaCl, 2 mM EDTA pH 8, 20 mM Tris HCl pH 8, and protease inhibitor cocktail). Then, all samples were precleared with 50 µL of protein G agarose/Salmon Sperm DNA beads (Millipore) during 3 h at 4 °C and centrifuged. Supernatants were incubated using anti-HA (Cell Signaling), anti-TCF-4 (Abcam, Cambridge, UK), or anti-IgG (Santa Cruz Biotechnologies, Dallas, TX, USA) rabbit antibodies overnight at 4 °C. Further, 50 µL of protein G agarose/Salmon Sperm DNA (Millipore) was added and incubated during 3 h at 4 °C. Samples were centrifuged, and the beads were washed four times with Wash Buffer I (1% Triton X-100, 0.1% SDS, 150 mM NaCl, 2 mM EDTA pH 8, 20 mM Tris-HCl pH 8, and protease inhibitor cocktail) and once with Wash Buffer II (1% Triton X-100, 0.1% SDS, 500 mM NaCl, 2 mM EDTA pH 8, 20 mM Tris-HCl pH 8, and protease inhibitor cocktail). Immunoprecipitated complexes were eluted with the Elution Buffer (1% SDS, 100 mM NaHCO_3_) and de-crosslinked with 200 mM NaCl for 5 h at 65 °C. All samples were treated with RNAse (200 µg) and Proteinase K (160 µg). DNA fragments were obtained using the phenol/chloroform protocol. Further, qPCR was performed to evaluated proteins interaction with *Sp5* promoter using specific primers: Forward 5′-GGGTCTCCAGGCGGCAAG-3′ and Reverse 5′-AGCGAAAGCAAATCCTTTGAATCC-3′. To analyze the data, the fold enrichment method was performed.

### 4.13. Proliferation Assays

C33A cells were seeded in a 60 mm dishes and transfected with 3 µg of E6 and β-catenin expressing plasmids, as indicated. After 24 h post-transfection, cells were harvested and seeded in a 96-well plate for 72 h. The MTS (3-(4,5-dimethylthiazol-2-yl)-5-(3-carboxymethoxyphenyl)-2-(4-sulfophenyl)-2*H*-tetrazolium, inner salt) assays were performed using the CellTiter 96 Aqueous One Solution Cell Proliferation kit (Promega, Madison, WI, USA), according to the manufacturer’s instructions.

For crystal violet assays, cells were fixed with 10% of formol/PBS for 30 min at room temperature while shaking. Cells were then stained with crystal violet/PBS for 15 min. After several washes, cells were treated with acetic acid/PBS and then measured at 490 nm. The data were graphed to determine the percentage of cell proliferation for each assay condition.

### 4.14. Statistical Analysis

Data showing the effects of HPV-18 E6 and E6*I and HPV-16 E6 on TCF-4 in the different assays are presented as mean ± SD. *p* was calculated by Student’s *t*-test or ANOVA Tuckey’s post-hoc analysis. Significance differences were accepted at *p* ≤ 0.05, as indicated.

## Supplementary Materials

Supplementary materials can be found at http://www.mdpi.com/1422-0067/19/10/3153/s1.
