# Supplementary material for: HPV-18 E6 Oncoprotein and Its Spliced Isoform E6*I Regulate the Wnt/β-Catenin Cell Signaling Pathway through the TCF-4 Transcriptional Factor
Source: Int J Mol Sci. 2018 Oct 13;19(10):3153. doi: 10.3390/ijms19103153 (PMC6214013; doi:10.3390/ijms19103153)
Supplement: Supplementary file 1 [file ijms-19-03153-s001.pdf]

Supplementary Material

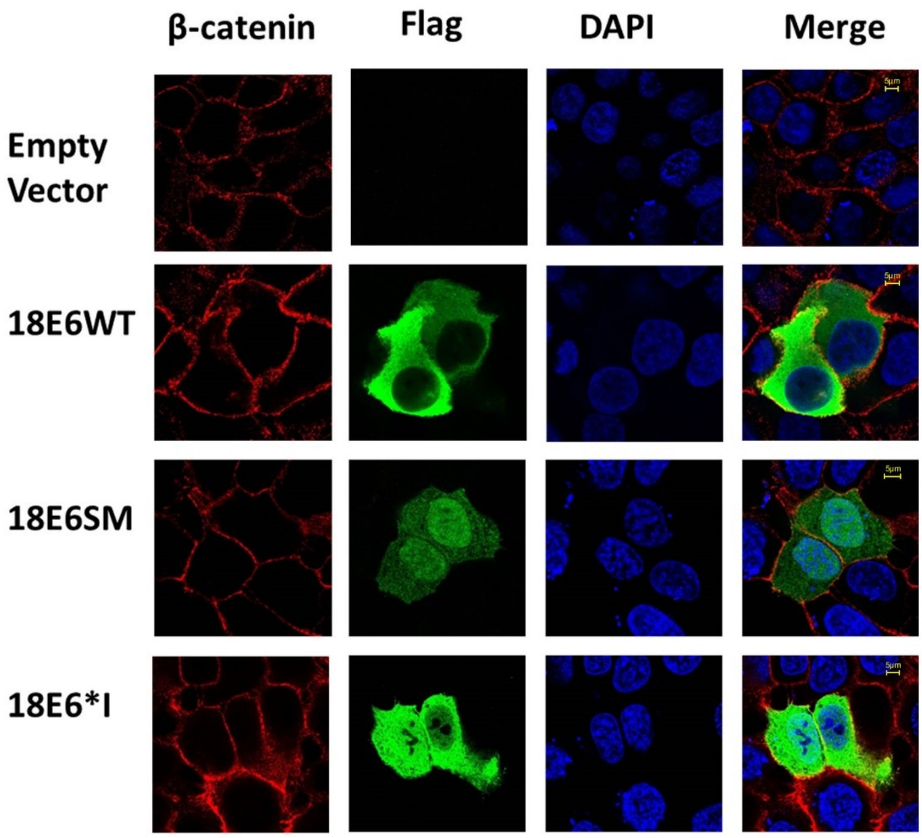

Figure S1. E6 and E6*I do not alter β-catenin subcellular distribution in immortalized keratinocytes. HaCaT cells were transfected with E6 expressing plasmids as indicated. 48 hours post-transfection cells were fixed and stained using specific antibodies to detect anti-Flag (Green) and anti-β-catenin (Red). Cells were also stained with DAPI (Blue) for nuclei detection. Images were acquired by confocal microscope. Data from three independent experiments were collected with a 63X objective oil immersion lens. Scale bar size 5μm.
